# Apicoectomy—The Culmination of Perfect Root-Canal Operations

**Published:** 1920-02

**Authors:** S. Sidney Gross


### APICOECTOMY—THE CULMINATION OF PERFECT ROOT-CANAL OPERATIONS

BY S. SIDNEY GROSS, D.D.S.

In spite of the advancement and remarkable achievement in dental science, the profession has been made the target of criticism by members of its own as well as by members of the allied medical profession. Their criticisms have been based upon the assumption that infected root-canals and morbid conditions about the apical ends of teeth are detrimental to the health of the patient, and are frequently the primary cause of systemic disturbance. It has also been stated that the present accepted method of treating and filling root-canals has proven unsuccessful, and that the only solution to the problem is extraction. This has been very clearly demonstrated by Professor Rosenow, of the Mayo Foundation, in his recent lecture before the members of the First District Dental Society of the State of New York. He stated very frankly and openly that it was a grave injustice to the patient to treat infected root-canals of those suffering with any systemic infection; that the only time it was permissible to treat devitalized teeth was in the absence of any constitutional disturbance, and with the patient's resistance rather high. Furthermore, that these root-canal operations should be performed under strict surgical asepsis, and that the root-canals should not be filled until the proper tests had been made to determine whether or not the canals were *absolutely sterile.* This naturally would demand of the dental surgeon a more thorough knowledge of bacteriology.

Whether or not a devitalized tooth is to be retained in the mouth of a patient depends mainly on (1) the constitutional condition of the patient, and (2) pathological conditions attending such a tooth. As has been previously stated, in the light of our present bacteriological knowledge, the saving of devitalized teeth is contra-indicated in patients suffering from any systemic disturbance. An attempt may be made at saving the tooth, providing it is followed by root resection only in such patients as are free from any systemic disturbance, whose resistance is normal, and if none of the following oral pathological conditions are found: (1) acute alveolar abscess with or without a fistula; (2) granuloma; (3) exostosis; and (4) foreign bodies in root-canals. The above conditions are absolutely essential, since we have come to realize the importance of sterilization of root-canals and of the areas above the apices of such teeth. While it may be possible to sterilize the root-canal, nevertheless, no matter how perfectly the technique may be carried out under strict asepsis, an element of doubt always enters whenever it comes to the region above the apex, and it is from this source that systemic infection follows. Therefore, to remove that element of doubt and make the operation successful, apicoectomy or root resection should be the culmination of all perfect root-canal operations.

The next important consideration in this work, assuming that we have a favorable patient for this operation, is the proper root-canal technique, performed with the greatest amount of skill and care and under ideal conditions. Then, also, it becomes imperative that proper roentgenograms of the involved region be obtained to determine the proper diagnosis. It should be understood, however, that the roentgenograms are primarily an aid to the diagnosis. The basic principles of the latter involve a physical examination in all its aspects. This operation is applicable to all the teeth excepting the third molars. Single-rooted teeth offer the least amount of difficulty; in multi-rooted teeth the operation is very often materially simplified by excising the entire root involved.

The diagnosis of an acute alveolar abscess is easily made from the clinical picture of acute suppuration. The invading pyogenic bacteria (of the streptococcus group) of high virulence cause a rapid and violent periapical tissue reaction, followed by pain, tenderness and swelling. The roentgenogram may show the destruction of tissue and bone. Sometimes it is quite distinct, having an irregular outline, whereas, on the other hand, it may appear like a faint veil, possibly due to the infiltration with pus of the cancellous structure. If the infection is hidden behind or in front of the root, it may not be evident on the film. The organism may be so virulent as to cause destruction within twenty-four hours after suppuration commences.

The diagnosis of a chronic alveolar abscess or granuloma is ofttimes difficult, particularly in the absence of any definite history and of objective as well as subjective symptoms. Such aids as history of previous subacute attacks, evidence of old fistulous scars, tenderness to digital pressure of the glands or lymph nodes of the neck, tenderness of the tooth to sharp percussion, and the presence of general systemic involvement, are valuable in arriving at the proper diagnosis.

A granuloma or chronic proliferative pericementitis may exist for many years, constantly causing a slow disintegration of the bone, and the cavity thus produced acts as a reservoir for a purulent exudate, without any external manifestation.

Cystic conditions may be diagnosed by the somewhat regular and definite outline and fairly dark shadow in the roentgenogram. As has been noted before, they are attended by necrosis, and can be seen near the surface of the bone at times as a very dark shadow varying in size from a pea to that of a plum.

### INSTRUMENTARIUM

The selection of the proper instruments necessary to carry out the operation of apicoectomy speedily and most successfully seems to be a matter of personal equation. The writer's clinical experience warrants the recommendation of the following instruments for this work: (1) Fischer syringe with proper needles for anesthesia. (2)

Numerous wooden applicators with sterile cotton twisted on one end. (3) Cheek retractors. (4) Sharp scalpel. (5) Periosteal separator. (6) Gum retractor. (7) Curved scissors. (8) Bone chisels or gouges of various sizes and shapes. (9) Nickel-weighted mallet. (10) Curets of various sizes. (11) Hemostats, straight and curved. (12) Full curved suture needles. (13) Needle holder. (14) Black silk No. 3. (15) Normal saline solution. (16) Novocain-suprarenin tablets E. (17) Tincture of iodin 7 per cent. (18) Sterile plain gauze. (19) Iodoform gauze. (20) Several pairs of thumb forceps.

In order to insure success in this operation, all canals should be properly sterilized and filled by a technique embodying all the principles of Rhein, Callahan and others. It is preferable to have this work done several days before attempting apicoectomy. The completion of all restorative work for the tooth in question should also be accomplished before this operation. Malocclusion should be corrected, and the mouth placed in proper hygienic condition.

### ANESTHESIA

Perfect anesthesia is absolutely essential to successful root-amputation. Either infiltration or conduction anesthesia may be employed. The clinical experience of the writer is that a combination of infiltration and conduction anesthesia undoubtedly gives the best results. It is imperative that the operation be performed with as little pain as possible, therefore any reasonable method of combination which accomplishes the desired result is suitable. If the correct technique is carried out the patient experiences practically no pain.

In operating on the upper six anterior teeth it is essential to make an infraorbital injection on both sides on account of the intermingling of the anterior peripheral branches of the superior maxillary division of the fifth cranial nerve. This procedure insures perfect anesthetization of the entire area. Two cc. of a 2 per cent. solution of novocain-suprarenin is all that is ordinarily required for a single injection. The application in the nostril of a 20 per cent. novocain-suprarenin solution in distilled water, applied with a good-sized nasal tampon (as suggested by Dr. Prinz) to paralyze the nerve filaments not reached by the injection is also advisable.

The time necessary to elapse between the injection and the first incision varies from ten to twenty minutes for tuberosity and infra-orbital injections, and one-half hour for mandibular injections. The beginning of an operation too soon causes the patient to suffer much unnecessary discomfiture and pain. It is unnecessary to hasten the first incision, since the anesthesia will last from forty-five minutes to one hour.

### TECHNIQUE OF THE OPERATION

The patient is prepared for the operation by having the head and eyes covered with sterile towels and the operating apron adjusted. A seven per cent. solution of tincture of iodin is applied to the mucous membrane around the field of operation. The lips are retracted, the saliva ejector is placed in the patient's mouth, and sterile pads are placed around the teeth. After observing the direction of the root in the roentgenogram, and its relation to the adjacent roots, the semilunar incision introduced by Partsch is made near the apex of the tooth in question, cutting deep through the periosteum to the bone. In both jaws the apex of the incision points toward the teeth. This permits the retraction of the formed flap without undue stretching of the adjacent parts. The periosteal separator is now used to disengage the entire flap from the bone. Old cicatricial tissue often causes difficulties in loosening this flap from the bony adhesions. This flap is held back by the assistant, using a gum retractor for that purpose. The bone over the affected root is carefully removed, using a mallet, sharp chisels and gouges, cutting out a good-sized oval-shaped window. The opening now reveals the infected root-end, which is severed with a small, sharp carpenter's chisel. This opening is enlarged with a curved chisel, and every vestige of necrosed bone and granuloma is scraped with curets from the periapical space until a clean, bony cavity is obtained. This bony cavity is then swabbed with warm normal saline solution, dried with sterile gauze, and inspected to determine whether any portion of the operation has not been properly performed. The area must be as clean as marble, and the filling in the canal of the resected root must be seen clearly. The sharp edges of the root stump are rounded off with chisels, and the cavity again swabbed with normal saline solution.

The successful termination of this operation is dependent upon the painstaking curettage of the fungoid growth from the bony cavity. The retractor is removed, the cavity is then painted with a seven per cent. solution of tincture of iodin, and in a great majority of cases the wound closed by saturating the flap with three sutures, using the No. 3 black silk. In suturing care should be taken to engage enough tissue to prevent tearing out of the sutures, and not to draw them too tightly.

### POST-OPERATIVE TREATMENT

This consists of advising the patient to apply cold compresses to the face over the area operated upon for five minutes every hour for the first day after the operation. If the post-operative pain is severe, the patient is advised to take five grains of aspirin every two hours, or ten grains every four hours until relieved. The patient should report every day for five days, and the operated area be touched with a little tincture of iodin. On the fifth day the sutures may be removed, using for this purpose a pair of thumb forceps and sharp scissors.

As has been stated before, all teeth are amenable to this operation excepting the third molars. Nevertheless a word of caution is necessary, as it will be found occasionally that the upper canines, bicuspids and first molars lie in close proximity to the maxillary sinus, and therefore care should be taken not to accidentally open into it. In the lower jaw the surgeon should pay attention to the mental foramen in the region of the apices of the lower bicuspids. The lower molars offer many difficulties on account of the heavy buccal plate, and the close proximity of the apices to the inferior dental canal. In such teeth it is preferable to remove the root *in toto.*

As to how long it will take nature to regenerate new bone, is a question that can not be answered at this time, since many factors concerning the regeneration of tissue are dependent upon the latent powers of the individual concerned.—*Dental Cosmos.*

